# How do patients and families evaluate attitude of psychiatrists in Japan?: quantitative content analysis of open-ended items of patient responses from a large-scale questionnaire survey

**DOI:** 10.1186/s12888-023-04732-w

**Published:** 2023-04-14

**Authors:** Ikuko Natsukari, Mari Higuchi, Tai Tsujimoto

**Affiliations:** 1 Yakitsubeno-Michi Clinic, 162 Nakazato, Yaizu City, Shizuoka 425-0014 Japan; 2 Faculty of Humanities and Human Sciences, Hokkaido University, Kita 10, Nishi 7, Kita-Ku, Sapporo City, 060-0810 Japan; 3 Nanzan University Institute for Social Ethics, 18 Yamazato-cho, Syowa-Ku, Nagoya City, Aichi 466-8673 Japan

**Keywords:** Attitude of psychiatrists, Evaluation by patients, Open-ended items, Quantitative content analysis, KH Coder

## Abstract

**Background:**

Patient and Public Involvement (PPI) has been widely advocated in psychiatric fields. In Japan, however, PPI has not been implemented in clinical practice. In order to improve quality of psychiatric service in Japan, it is essential to understand psychiatrists’ attitudes from the patients’ perspective as a first step in practicing PPI. This study aimed to investigate the patients’ evaluation of psychiatrists’ attitudes by illustrating themes appeared in the questionnaire survey.

**Methods:**

This study used the data obtained from the questionnaire survey responded by 2,683 patients with family members who belong to the family associations for psychiatric patients in Japan. Three open-ended question items in this survey, "criteria for selecting a psychiatrist (784 patient responses, response rate 29.2%),” “attitude of the psychiatrist in charge (929 patient responses, response rate 34.6%)," and "communication skills of the psychiatrist in charge (739 patient responses, response rate 27.5%)" were analyzed by co-occurrence network using KH Coder software.

**Results:**

The common theme observed in all three items was whether psychiatrist took sufficient consultation time. The criteria for selecting a psychiatrist were summarized whether psychiatrist provided appropriate advices for patients’ problems, whether psychiatrist cared about patients’ demands and whether psychiatrist informed to patients about diseases and medications. The attitudes of the psychiatrists in charge that patients had most wanted their psychiatrists to improve were: psychiatrists only watch the computer, make diagnosis according to the patients’ individual condition, and try to build a relationship of trust with the patient. The patients’ demands regarding communication skills of the psychiatrist in charge included: whether the psychiatrist communicated in a way that improves the patient's psychological state, whether the psychiatrist was attentive to the patients’ family, and whether the psychiatrist could control his/her own mood during the consultation.

**Conclusion:**

The results reflected the patients’ demands that do not appear in closed-ended items. It was suggested that patients’open-ended responses to questionnaires and their involvement in the psychiatric research (PPI) may provide more insight into improving pshchiaric care in Japan.

## Background

### Global trends of Patient and Public Involvement in the medical research

In recent years, Patient and Public Involvement (PPI) in medical research has become a worldwide recommendation. An overview of PPI can be represented by following two studies; one is that Truman [1] pointed out the importance of patients’ participation in the clinical research, the another is that Lloyd [2] proposed democratizing medical researh. Following these research streams, National Institute for Health Research (NIHR) defined the PPI in medical research as “research being carried out ‘with’ or ‘by’ members of the public rather than ‘to’, ‘about’ or ‘for’ them” [3]. To acheive PPI, researchers have to work and collaborate with patients and/or members of public at all stages of the research process, from planning to implementation [3].

Turner [4] points out that the origin of the PPI lies in patients' dissatisfaction with conventional research that does not reflect their experiences and the momentum for patients themselves to participate in research to bring their experiences to bear on research and medical care. Against this backdrop, an increasing number of studies are using "Patient-Reported Outcomes: PROs," in which patients are asked about their experiences with and dissatisfaction with health care services. PROs can be considered one of the efforts of PPI. Kingsley et al. [5] noted the information from the PROs has many uses, including quality improvement of research and treatment, economic evaluation, and provides important feedback to health care providers allowing them to compare their clinical care. In psychiatric research, PROs such as patient satisfaction have tended to be concerned with patients' evaluative abilities and insight [6], but in recent years they have proven to be valuable predictors of the quality of health care services received by patients [7, 8].

### PPI in Japanese Psychiatric Research

In Japan, the Agency for Medical Research and Development [9] and Japan Pharmaceutical Manufacturers Association [10] have issued a statement on PPI. The Japan Pharmaceutical Manufacturers’ Association is promoting development under the theme of “Drug Development Utilizing the Voice of Patient” [10]. In this trend, the importance of PPI, including PROs, are being recognized, however, the number of study adopting PROs is still limited.

Yamaguchi et al. [11] conducted systematic review of studies including PROs related to patient-psychiatrist relationshps, communication, and decision making in Japanese psychiatric practice, and they pointed to the paucity of large surveys as research evidence issues. As one of the representative examples of the PROs studies in Japan, Ishii et al. [12] examined the efficacy of shared dicision making using the patients’ self-reported satisfaction at discharge, however, their sample size was limited to 58 inpatients at one psychiatric hospital. In addition, a new psychiatric registry was launched to promote multicenter collaborative research aimed at elucidating the pathophysiology of psychiatric disorders and developing new diagnostic and therapeutic methods [13]. Although many of these studies employ electrical patient-reported outcomes (ePRO), which are also included in the PROs, patients primarily rate symptoms such as Positive and Negative Affect Schedule (PANAS) [14] and do not adopt patient evaluations of medical services.

Thus far, Japan has not conducted a large-scale survey on PROs, and PPI in psychiartic research is still in its infancy compared to other countries. Given the state of psychiatric care in Japan, about half of all psychiatric inpatients experience involuntary hospitalization [15]. In addition, even in the case of voluntary hospitalizations, it is reported that 54% of these patients spend the entire day in a closed ward [16]. Because of the high likelihood of involuntary hospitalization, patients may be less likely to express their opinions to psychiatrists, and health care providers may be more likely to disregard incorporating patients' evaluations of their health care services, which may inhibit researchers’ motivation to increase PPI.

### A first large-scale survey for PPI attempt in Japan

In response to paucity of large survey adopting PPI in Japan, in 2015, the first author conducted a survey on how patients and families evaluate the attitude of psychiatrists (“Questionnaire on psychiatrists’ examination skills, attitudes, and communication skills”). Before conducting this survey, the first author publicly announced her position as a psychiatrist, psychiatric patient and a family member of the schizophrenic patient [17]. This coming out has led the first author to hold a total of 250 roundtable discussions and lectures with patients and their families throughout Japan. In the course of these discussions, and patients and ther families had requested to incorporate more “patient or family perspectives” into psychiatric care and research. Therefore, 15 representatives of patient and family associations participated in the planning of this questionnaire survey, and all questions were developed based on the input from patients and families. Furthermore, to ensure that the opinions of patients and families who did not participate in the survey design were not left out, the survey included not only closed-ended questions, but also open-ended questions that will be analyzed in this paper, as described next.

This was the largest and first survey as PPI ever conducted in the filed of psychiatry in Japan. The results of the analysis of closed-ended questions in this survey which employed Likert-style were publised in 2018 [18]. In the previous study [18], the results showed that patient respondents were generally satisfied with most of their psychiatrist’s attitudes described in the closed-ended items; 85.1% of them agreed with the item of “my doctor in charge looks me in the eye and face,” 82.5% of them agreed with the item of “my doctor in charge is reliable” and 84.3% of them agreed with the item of “my doctor in charge listens to me well”.

However, it has been pointed out that there is a bias of tacit obedience tendency towards selecting positive responses for closed-ended items adopting Likert-style items [19]. Another limitation of closed-ended questions, the scope of what the researchers can investigate on patients’ thoghts and requirements toward psychiatrists must be limited to the set of choices presented in each questions and we can not clarify respondents’ requests which we had not anticipated in advance. Considering these pitfall of the responses to closed-ended questions using Likert-style questions, the questionnaire of this survey was designed with each closed-ended questions followed by an open-ended questions. In these open-ended qeustions, patients and their families were asked for their requests that were not included in the options for closed-ended questions.

Based on the results obtained from open-ended questions, this study disccuses what are needed for future practice of psychiatrists to meet patient deamands, and also discusses the possibility of using PROs in research as part of the promotion of PPI.

## Methods

### Summary of the data

In this study, we used the text data of responses to three open-ended questions in the survey “Questionnaire on Psychiatrists’ Examination, Attitude and Communication Skills” [18]. Before describing the method for the analysis of the open-ended questions, a brief overview of the data in this survey is provided.

In 2015, 18,000 unmarked self-administered questionnaires were mailed to members of the National Federation of Associations of Families with the Mental Illness in Japan Minna-net, the Community Mental Health & Welfare Bonding Organization, and related offices, and 6,341 people responded^1^. Of these, 6,202 valid responses were received (2,683 from patients and 3,519 from family members). In order to focus on the patients’ opinions, only 2,683 patient respondents were included in this study. Most of the patients were in their 30s and 40s, and most of their family members were in their 60s and 70s. 52.0% of the patients and 24.4% of their family members were male. The most common place of hospitalization was a psychiatric hospital or clinic, and the most common illness was schizophrenia (71.6%) [18].

### Quantitative content analysis of open-ended questions

Lofthus et al. [20] and Lovell et al. [21] conducted surveys on patient satisfaction with overall medical care using a combination of a questionnaire survey adopting closed-ended questions and qualitative research. They reported that, by using qualitative research in addition to evaluating patient satisfaction using the closed-ended questions, they were able to discover patient intentions that could not be extracted using the closed-ended questions only. In orderd to realize extracting patients demands for psychiatrists which are not included in the closed-ended questions prepared by the researchers, this study analyzed three open-ended items in the questionnaire survey.

The open-ended questions used in the analysis were: “If you have any other points to refer to regarding the selection criteria of physicians, please feel free to fill in here,” “If you have any other impressions regarding the ‘attitude’ of your doctor in charge, please fill in here,” and “If you have any other impressions regarding the ‘communication skills’ of your doctor in charge, please fill in here.” With using these data, this study exploratively reveals the criteria for psychiatrist selection and the patients’ demands regarding their psychiatrist's attitude towards patient examination and communication skills, which were not appeared in the closed ended questions^2^, through a quantitative content analysis of open-ended items.

Quantitative content analysis is a method of organizing or analyzing text data using quantitative methods based on content analysis [22, 23]. Specifically, text data such as sentences of responses to each open-ended item are broken down into words, counted, and the frequency of occurrence of words and the connections between words (co-occurrence relationships) are calculated using statistical methods. Quantitative content analysis incorporates the qualitative meaning of words in the context of the original data and the analyst’s interpretation of that meaning into the analysis process. By adopting quantitative and qualitative methods cyclically [22, 23], first, it is possible to analyze all responses to open-ended question items’ data in a large-scale questionnaire survey. Second, the analysis results can reflect a qualitative interpretation of the meaning in the context of the word’s original data. As a result, it is possible to present the qualitative content of the patient’s demands systematically noted in the responses to open-ended questions.

 This study uses KH Coder, a software developed to implement the method of quantitative content analysis described above. It is very important to note that the KH coder can reproduce the results shown in the present study. The KH Coder, which now supports a variety of languages, is well suited for this study because it was originally designed to analyze the Japanese language, which was difficult to analyze quantitatively.

### Analysis process

The analysis process used was as follows. First, a dataset of patient responses was created as an Excel file and subsequently loaded into KH Coder, focusing the analysis on valid responses for all attribute items of respondent’s position, gender, age, and diagnosis. Second, for each open-ended item response, a co-occurence network was created to extract themes contained in the patients’ responses. Co-occurence networks have been used early on the field of quantitative content analysis to investigate themes or topics that appear in text data by looking for groups of words with similar occurence patterns [24]. But before getting co-occurence networks, we removed the word “think” from the analysis. This is because if commonly used words that are not directly related to the question were included in the analysis, when analyzing the co-occurrence relationship between words, topics that are not originally related to each other might be connected [22, 23]. In addition, in order to extract the themes expressed in the responses to each open-ended question item from the co-occurrence network, we used KWIC Concordance to search the original data for specified words to see their meaning in the original data. Through these processes, we analyzed the themes appeard in patiens’ responses in criteria for selecting the psychiatrist, as well as the patient’s demands regarding the psychiatrist’s examination attitude and communication skills.

**Fig. 1:**
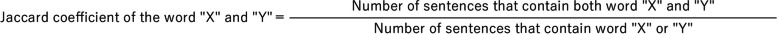
Jaccard Coefficient ^1)^ The authors created this formula based on Higuchi [25] ^2)^ The "sentence" means each respondent's answer in this paper

The lines connecting words in the figures of co-occurrence network were drawn based on Jaccard coefficients. The Jaccard coefficient is the value obtained by the formula shown in Fig. 1 and represents the ratio of the number of responses in which words A and B occur simultaneously (co-occur) to the total number of responses in which either word A or B occurs. Its value ranges from 0 to 1. The larger this coefficient, higher the number of responses in which word A and B were simultaneously used. In the co-occurrence network, the higher the Jaccard coefficient, the stronger the co-occurrence relationship, and the darker the color of the line connecting words [22, 23, 25].

In this co-occurrence network, the size of the circle surrounding a word indicates the number of occurrences of that word. The larger the circle, the more often the word appeared in the responses. However, a word with a large circle does not necessarrily mean something important. For example, commonly used words such as “doctor” had a large number of occurrences in each item; consequently, this word was surrounded by a large circle. In the co-occurrence network, we checked not only the number of occurrences, but also the co-occurrence relationship and the meaning of the words in their original context. The themes in the responses were subsequently categorized and surrounded by a dashed line, with names representing their contents. The open-ended items used in this study were formatted to ask patients’ demands that were not listed in closed-ended items adopting Likert-style presented earlier in the questionnaire. Therefore, the responses of open-ended items are complementary to the closed-ended items.

## Results

### Respondent demographics

Table 1 shows the basic attributes of the respondents with respect to the criteria for psychiatrist selection, the attitude of the psychiatrist in charge, and the communication skills of the psychiatrist in charge. The number of responses and response rates differed among three items: 784/2,683 responses (response rate: 29.2%) for "criteria for selecting a psychiatrist, " 929/2,683 responses (response rate: 34.6%) for "attitude of the psychiatrist in charge" and 739/2,683 responses (response rate: 27.5%) for "communication skills of the psychiatrist in charge. The largest number of respondents in all categories were in their 30s and 40s, and suffered from schizophrenia. With regard to the gender of the respondents, slightly more number of women than men responded only to the item assessing the “attitude of the psychiatrist in charge.”

**Table 1:** Frequencies of patient responses to open-ended items

	Psychiatrist selection criteria (*n* = 784, response rate: 29.2%)	Attitude of the psychiatrist in charge (*n* = 929, response rate: 34.6%)	Communication skills of the psychiatrist in charge (*n* = 739, response rate: 27.5%)
Gender			
Female	359	472	362
Male	425	457	377
Age classification			
Under 20s'	52	54	41
From 30s' to 40s'	454	566	450
From 50s' to 60s'	264	299	236
Over 70s'	14	10	12
Diagnosis group			
Schizophrenia group^a^	487	581	469
Bipolar disorder group^b^	189	216	163
Other group^c^	96	121	98
Non-disclosure	12	11	9

^a^Including schizophrenia and schizoaffective disorder

^b^Including major depressive disorder, manic and bipolar disorder

^c^Including anxiety disorder, panic disorder, specific phobia, obsessive–compulsive disorder, PTSD, feeding and eating disorders, personality disorders, Neurodevelopmental Disorders and seizure disorder

The following section focuses on themes related to patient demands not listed in the closed-ended items using Likert-style. The results for each item were written in brackets after the citation of the response of the patient, with the responder’s attribute written as (age-group, gender, diagnosis group).

### Psychiatrist selection criteria

**Fig. 2:**
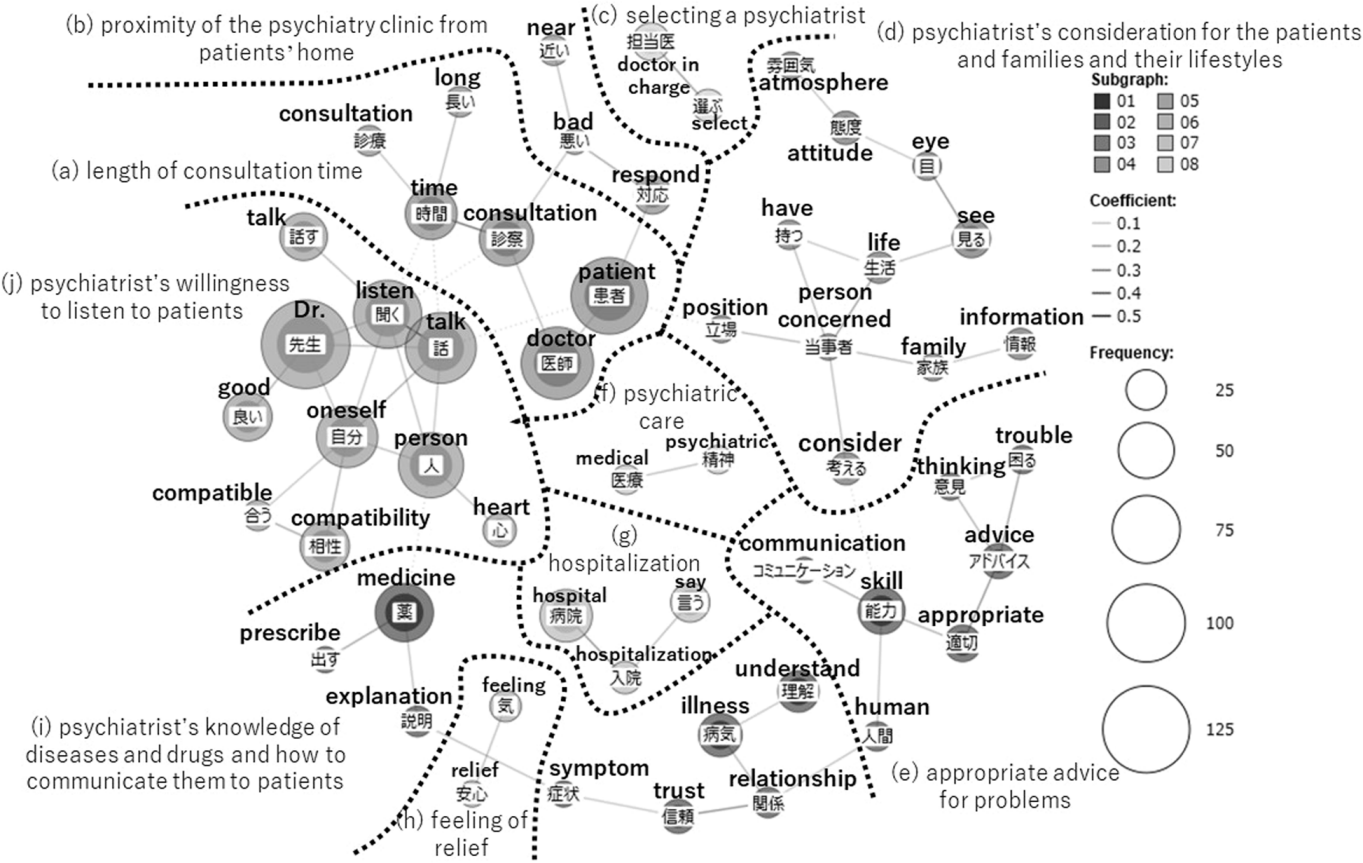
Co-occurrence network of the open-ended item “psychiatrist selection criteria”

A co-occurrence network was created from the responses regarding psychiatrist selection criteria (Fig. 2), and the themes of the responses were divided into groups (a) – (j). The dashed lines in the figure were drawn to make the themes easier to understand (the same applies to the “attitude of the psychiatrist in charge” and the “communication skills of the psychiatrist in charge” in the following sections). The following paragraphes describe the main groups in Fig. 2: (a) length of consultation time, (j) psychiatrist’s willingness to listen to patients, (d) psychiatrist’s consideration for the patients and families and their lifestyles, (e) appropriate advice for problems, (i) psychiatrist’s knowledge of diseases and medications and how to communicate them to patients and (b) proximity of the psychiatry clinic from patients’ home.

First, the co-occurrence of “time” and “consultation” in the group (a) showed whether the psychiatrist took a long time to examine the patient, such as “ (the important criteria for me are) length of consultation time and whether he or she listens carefully” (by a respondent in 30s, female, bipolar disorder group)^3^.

The group (j) was a theme related to the dialogue with the psychiatrist during the medical examination, and “talk” co-occurred with “listen.” The respondents mentioned whether the psychiatrist listened to the patients or not, as in the example of “a doctor who listens to me well” (respondent in 50s, male, schizophrenia group).

The group (d) was a theme that required psychiatrists to put themselves in the shoes of patients and their families, with “person concerned” co-occurring with “life,” “consider,” “position,” and “family.” “Whether the doctor will consider together with the person concerned, on the plan of care” (respondent in 30s, male, schizophrenia group), “A doctor who puts himself or herself in the position of person concerned” (respondent in 20s, female, schizophrenia group), “Whether the doctor has a person concerned (person with psychiatric diagnosis) in his or her family member. I heard that my current doctor’s sister has schizophrenia, so I switched my doctor to that doctor” (respondent in 50s, female, bipolar disorder group). As for “family,” a few respondents asked for consideration for family members, such as “I wonder if they will listen to my family’s situation as well” (respondent in 40s, female, bipolar disorder group).

In the group (e), “advice” co-occurred with “appropriate” and “trouble.” For example, “I want someone who can give me appropriate advice and guidance on how to behave and think” (respondent in 50s, female, manic-depression group), “I want advice when I am in trouble. I want my doctor to understand my suffering” (respondent in 20s, male, schizophrenia group).

The group (i) was the theme of the psychiatrist’s knowledge of mental illness and its treatment, and the psychiatrist’s willingness to share and communicate his or her knowledge to the patient. First, “medicine” co-occurred with “prescribe” and “explanation.” Respondents requested that the medication be prescribed corresponding to their conditions and that they be provided a detailed explanation of the medication. These tendencies were seen in these responses: “A doctor who responds to my mental condition and prescribes me medicine” (respondent in 50s, female, manic-depression group) and “Whether or not the doctor gives me detailed explanations about medicine” (respondent in 40s, male, schizophrenia group). Next, in the co-occurrence of “illness,” “understand,” and “relationship,” we observed responses such as “Someone who understands my illness and can build a relationship of trust” (respondent in 40s, female, schizophrenia group), “Whether the doctor is kind, understands my illness, and tries to cure me. Whether the doctor tries to have a relationship with me where we can discuss anything” (respondent in 50s, male, other group). “Symptoms”co-occurred with “trust” and “explanation,” with specific responses such as “I do not trust a doctor who makes me wait for several hours and finishes examining me in five minutes. Also, doctors who treated patients as if it were their ‘job’ did not listen to my symptoms very well” (respondent in 20s, female, schizophrenia group), and “I go and see my doctor once a month. The doctor asks me, ‘How are you doing?’ but he or she does not ask me whether my anxiety or symptoms improved or not. Indeed, the doctor has never explained my medications and never changed it.” (respondent in 80s, male, bipolar disorder group). In terms of trust with the psychiatrist, patients valued their psychiatrists’ willingness to understand their illnesses and their explanations of symptoms and medications.

The group (b) was physical access to the clinic or the hospital. The co-occurrence of “near” and “bad” denoted the ease of access when the patient was unwell. Examples of specific responses with this co-occurence are “When I am feeling bad, I do not like to go out, so I prefer the clinic near my house” (respondent in 50s, female, bipolar disorder group) and “Near home so that the doctor will respond immediately to my sudden bad mental condition” (respondent in 40s, male, bipolar disorder group). On the other hand, as seen in the response “Not too near my house because it would be difficult to go if someone sees me, and not too far because it would be difficult to go to the hospital from my home” (respondent in 20s, female, schizophrenia group), a few respondents were worried that their neighbors would find out about their psychiatric hospital visits, and therefore, preferred a moderate proximity.

### Attitude of the psychiatrist in charge

**Fig. 3:**
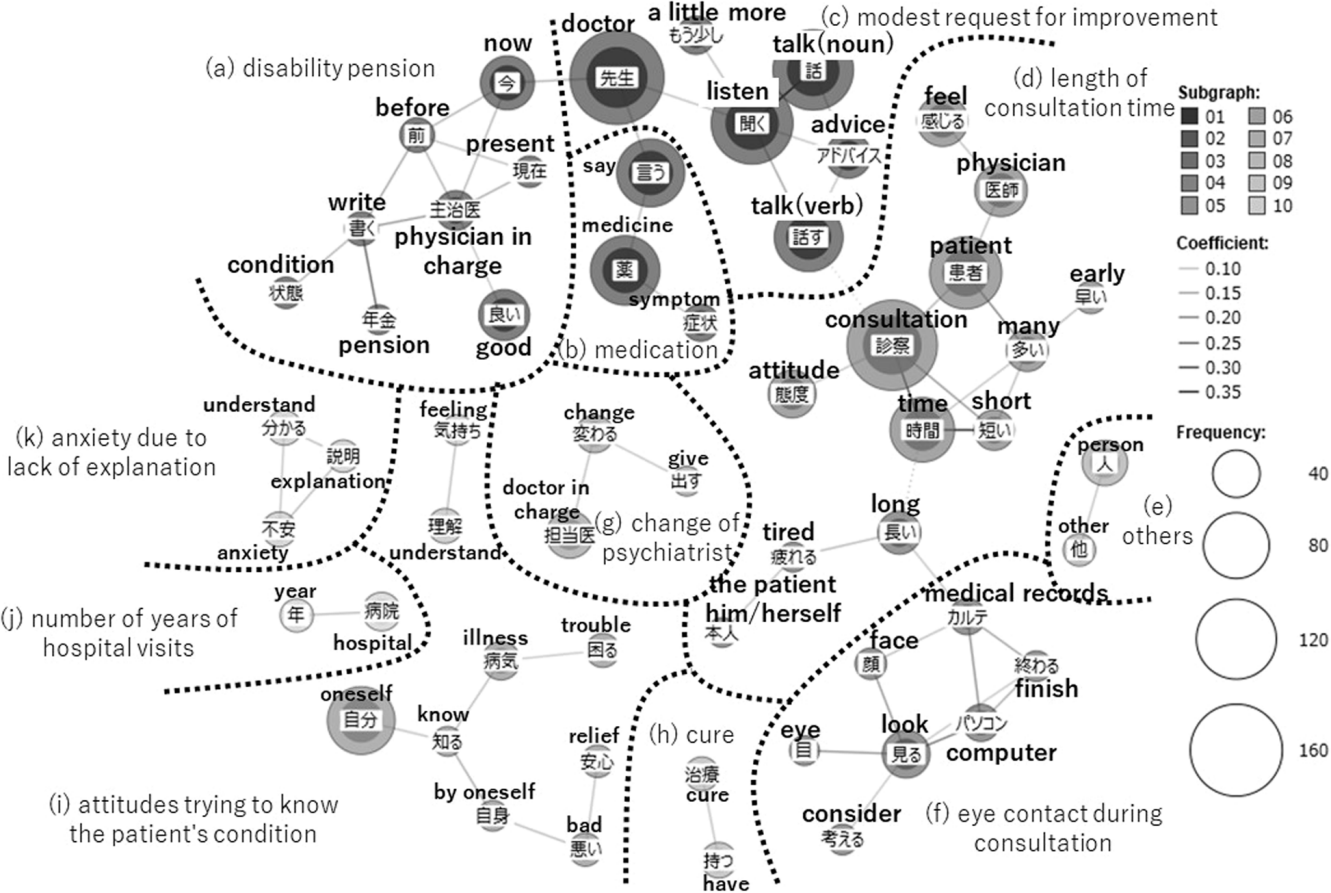
Co-occurrence network of the open-ended item “attitude of the psychiatrist in charge”

A co-occurrence network was created from the responses regarding the “attitude of the psychiatrist in charge” of the patient’s examination (Fig. 3), which was divided into the group (a)–(k). In the following sections, we focus on the group (d) length of consultation time, the group (f) eye contact during consultation, the group (c) modest request for improvement, and the group (a) disability pension.

First, in the group (d), a co-occurrence relationship was found between “consultation” and “time.” The word “consultation” appeared 154 times, second only to “doctor” (168 times), which had the highest number of occurrences. This makes the group (d) a topic of great interest to patients. The patients were concerned that their psychiatrist did not spend enough time for the examination. These are examples of the specific responses of this co-occurence: “The clinic I went to before, the average waiting time to take consultation was 3.5 h. I went once every 3 weeks, I had to wait up to 6 h and 50 min. I couldn’t even talk one-fifth of what I wanted to talk about, and the doctor tried to cut me off by creating an intimidating atmosphere” (respondent in 30s, male, schizophrenic group) and “When I told the doctor my symptoms to respond to his question ‘How are you?,’ the doctor said, ‘The medicine is the same as before,’ and the doctor shortened the consultation time” (respondent in 30s, male, other group).

In the group (f), “computer” co-occurred with “look,” “medical record,” and “finish”. Specific responses of this co-occurence included, “I am bothered by the fact that my doctor only looks at the computer screen” (respondent in 30s, female, schizophrenia group), and “My doctor listens to me while typing medical records on the computer, so I want him or her to type after we finish talking” (respondent in 30s, female, bipoplar disorder group). Patients expressed frustration that their psychiatrist only looked at the computer and not at the patients’ faces, during the consultation.

In the group of (c), the words “a little more” co-occurred with “listen.” Examples of this co-occurence include “The consultation time is short. It takes about 3 min per person. The doctor tries to end the consultation early. I wish the doctor would listen to me by taking a little more time” (respondent in 30s, male, schizophrenia group) and “I know there are limits of consultation to what can be done at a university hospital, but I would like to have a little more concrete advice and to be listened to a little more concretely” (respondent in 40s, female, schizophrenia group). The patients were not overtly dissatisfied with their psychiatrist’s attitude during the consultation, but they were not fully satisfied and wanted improvement.

The group of (a) was “disability pensions.” Specific responses of this theme included “Regarding obtaining a disability pension, I was still in the process of going through the procedures, and despite my complaining that I was very sick and I could not work, my doctor was relentlessly reluctant even though it was not a loss for the doctor” (respondent in 40s, female, bipolar disorder group). “Pension” co-occured with “writing,” in responses such as “I have not yet had my current doctor write a medical certificate to obtain a disability pension, but when I had my previous doctor write it, my disability level was underestimated. I still do not know what was written in the certificate, and I do not understand why my level has been lowered” (respondent in 50s, female, bipolar disorder group). Regarding disability pensions and writing a medical certificate, the Likert-style closed-ended items presented earlier in these open-ended items asked, “Will your doctor write a careful medical report for you to obtain a disability pension?” In spite of the existence of these question items, the fact that patients provided open-ended answers further suggests that they want their psychiatrist to write a proper medical certificate in accordance with their own condition at the time.

### Communication skills of the psychiatrist in charge

**Fig. 4:**
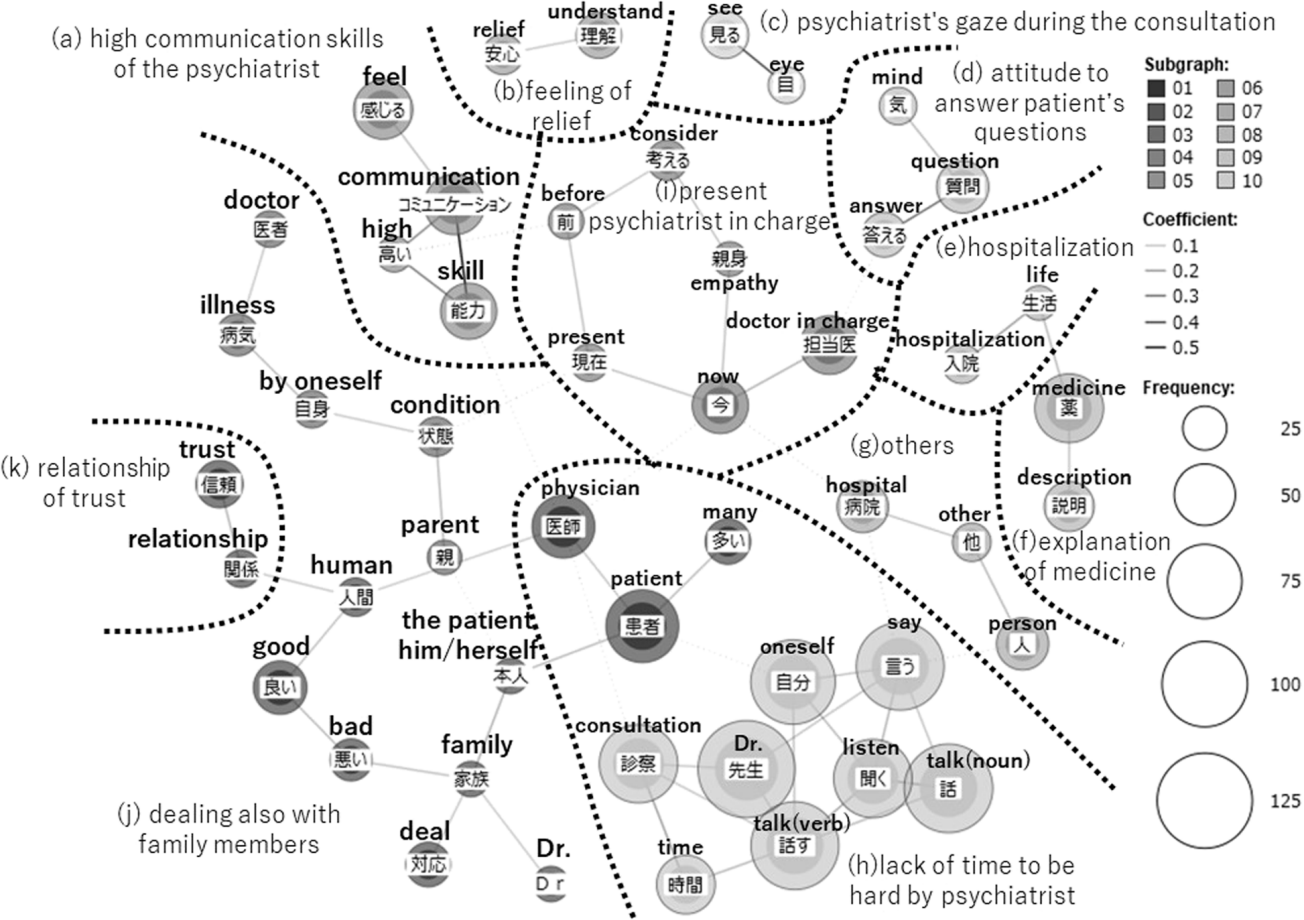
Co-occurrence network of the open-ended item “communication skills of the psychiatrist in charge”

A co-occurrence network was created from the responses regarding the “communication skills of the psychiatrist in charge” (Fig. 4), which was divided into the group (a)–(k). Here we address the following topics not mentioned in the previous two open-ended items: the group (a) high communication skills of the psychiatrist, the group (j) dealing also with family members, and the group (k) relationship of trust.

First, in the group (a), the word “communication” co-occurred with “high,” signifying a positive attitude of the psychiatrists. This co-occurence was observed in specific responses such as “I think the level of my doctor’s communication skills is high because he or she was able to make my long-standing ‘anger’ disappear just by seeing him or her” (respondent in 40s, female, bipolar disorder group), “My doctor’s communication skills are so high that I wonder if he or she has been specially trained. I have read Dr. XXX’s books, and I feel something very similar, in a good way. I can relax and talk to him or her” (respondent in 50s, female, bipolar disorder group), and “Greetings, standing up before examining me and saying, ‘Nice to meet you,’ and ‘Please take care of yourself,’ made me feel at ease. My doctor does not tell lies, so I can trust him or her. The treatment is specialized and difficult (for me to understand), but my doctor is kind enough to answer whatever I ask. The doctor in charge is able to respond in a way that fits my situation. I was always out of control, but the way my doctor treats me makes such a difference, so I am convinced that his or her communication skills must be high” (respondent in 30s, female, other group). In all of these responses, patients perceived a change in their own psychological condition and behaviors. Patients also attributed these positive changes to their psychiatrist’s good communication skills. However, co-occurring relationships between “communication” and “high” also included the following responses: “‘My communication skills are very high,’ my doctor said. However, that is about the ‘medical’ side. Mental disorders require cooperation among ‘medical,’ ‘psychological,’ and ‘welfare.’ I would like to see more clinical psychologists and psychiatric social workers” (respondent in 30s, male, bipolar disorder group) and “I think my doctor’s communication skills are very high because he or she empathizes with me and listens to me even when I cannot talk well, but even so, I sometimes distrust him or her. Because he or she will not let me out when I am admitted to a closed ward” (respondent in 40s, male, schizophrenia group). While evaluating the doctor’s communication skills as “high,” some respondents pointed out their dissatisfaction and distrust about the psychiatrist’s attitude other than communication.

In the group of (j), a co-occurring relationship between “condition” and “parent” was observed. Examples of this include: “My husband is also a patient. My doctor asks me about my husband’s condition and how we are feeling, and my doctor understands my parents and sister very well. 14 years have passed, and every day I wish I could have found my current doctor sooner” (responder in 50s, female, other group) and “When I was exhausted both mentally and physically from caring for my older parents, the doctor not only gave me advice to improve my own condition, but also helped me with how to proceed with my parents’ care” (respondent in 60s, female, bipolar disorder group). Patients felt relieved and satisfied that their psychiatrists paid attention to not only their own problems, but also their families’ problems.

While these positive evaluations were present, “family” co-occurred with “deal,” “bad,” and “patient himself or herself.” Examples of this include “My doctor does not teach me how to deal with family members or teach my family how they should deal with the patient himself or herself. He or she does not invite us to family meetings. He or she also does not tell us where we can meet to discuss things with other person concerned” (respondent in 40s, female, other group) and “There was a time when my symptoms got worse, and I complained about my anxiety and pain for a long time during the consultation. I suffered from suicidal ideation for several months, and I told my doctor several times during the medical consultation, and I think there were enough signs (there were also complaints from my family members). However, the doctor told me that I lacked the awareness of illness, and my family’s complaints were ultimately passed around, so my symptoms and complaints were overlooked. In order to handle a large number of patients, I feel that doctor deals with patients’ diagnosis rather than individual symptoms. I went to the hospital because I have no expertise, so it was hard for me to be told by my doctor that I just lacked awareness of illness” (respondent in 30s, male, other group). Patients were dissatisfied with the lack of outreach to their families from their psychiatrist in charge or the psychiatrists’ failure to listen to their complaints.

Another feature of the group (j) is the co-occurrence of “good” and “bad,” which indicated the psychiatrist’s mood. Some of the responses indicated that the patients felt stressed because the mood of their psychiatrist varied greatly with each visit: “The doctor’s mood fluctuates between good and bad” (respondent in 30s, male, schizophrenia group)”, “The doctor himself or herself is very depressed and I can easily tell when he or she is in a good mood and when he or she is in a bad mood. I am compelled to be sensitive to my doctor’s mood” (respondent in 50s, female, bipolar disorder group). Another respondent stated, “I wish they would tell me sooner if my illness is going good or bad” (respondent in 60s, female, schizophrenia group), requesting clear information on how the psychiatrist views the patient’s condition.

The co-occurrence of “trust” and “relationship” in the group (k) indicated a desire to build a trusting relationship with the psychiatrist in charge. “I have a very difficult time building a relationship of trust with my doctor because the primary doctor changes almost every year due to the university hospital system. I have other illnesses as well, so it is hard for me when my doctor tells me, ‘If you do not like my treatment plan, go to another hospital yourself.’ I just wanted my doctor to cooperate with other departments in terms of medication to treat my illnesses” (respondent in 30s, female, bipolar disorder group), and “I want my doctor talk to me and let me talk a lot with him or her about, for example, what kind of hobbies I have or how I spend my daily life. Doing so, we can build a relationship of trust” (respondent in 30s, male, schizophrenia group) were observed as responses of this theme. From these responses, it can be said that, remaining in charge of the patients and having an attitude of trying to get the patients to talk led to trust in their psychiatrist.

## Discussion

### Summary of results

The purpose of this study was to explore and extract patients’ deamnds that could not be captured by the closed-ended items using Likert-style by analyzing patients’ responses to open-ended question items, based on co-occurrence networks.

The authors found that the patients’ criteria for selecting a psychiatrist were length of consultation time, appropriate advice, ability to listen to the patient, consideration for the patient and family, general explanation of medications, and proximity to the patient’s home. As for the patients’ evaluation of psychiatrist’s attitude, they place importance on whether the psychiatrist has enough time for consultation, whether the psychiatrist looks at the computer only, and whether the psychiatrist prepares a diagnosis according to the patient’s physical condition. Regarding the patients’ evaluation of psychiatrist’s communication ability, they concerns whether the psychiatrist communicates with the patient psychologically, whether the psychiatrist cares about the parents, and whether the psychiatrist has a relationship of trust with the patient.

Further, the co-occurrence of “consultation” and “time” was common to all three responses. The responses of each patient to the open-ended question items show a strong desire for a longer duration of consultation. Thus, it can be said that consultation time was the most important concern for patients.

For it’s reason, following four points can be considred. Firstly, it is the Japanese medical fee system. Under the public medical fee system for psychiatric insurance treatment, psychiatrists can only bill for "outpatient psychotherapy" for consultations lasting more than 5 min but less than 30 min. The additional amount for consultations lasting more than 30 min is fixed at 700 yen, no matter how long the consultation takes [26]. Because of this system, the more time a psychiatrist spends on a consultation, the more he or she loses financially. Thus, in order to increase the effectiveness of treatment in a such short consultation time, they are obliged to focus on drug therapy in stead of taking more time on the consultation.

Secondly, it is the relatively low number of psychiatrists per population. The number of psychiatrists in Japan was 0.12 per 1,000 population in 2016, ranking 25th in the Organisation for Economic Co-operation and Development (OECD) [27]. In contrast to the world's highest number of psychiatric beds, the number of psychiatrists is not very large. In order to encourage more medical students to pursue a career in psychiatry, Japanese medical school education should incorportae practical training in community welfare and public health fields to develop a clinical sense of psychiatric care.

Thirdly, there is no primary care physician system. Since there is no primary care physician system in Japan, patients can visit any department at their own discretion and they are more likely to repeatedly visit a psychiatrist, even when it is unnecessary. Interplaying of these factors can have contributed to the inability of psychiatrists to take sufficient time with each patient until the patients feels to be fully examined by the psychiatrist, leading to greater demands on consultation time.

Fourthly, in Japan, there are not sufficient opportunities for psychiatrists to improve their communication skills with patients. Initial training for psychiatry residents does not allow sufficient time for patient interviews. In addition, once they become psychiatrists, they do not have enough opportunities to learn how to spend time interviewing patients and their families in order to gain experience in acute care and mandatory care.

To overcome these problems, as a first step, there should be training opportunities for psychiatrists and psychiatry residents to improve their communication skills so that they can talk to patients effectively in a short amount of time. As a second step, family physicians should be trained in basic psychiatric interviewing and diagnosis. It can be expected that the family physician system will prevent patients from becoming overly dependent on psychiatrists. As a third step, to creat a medical system that rewards psychiatrists for their time-consuming and specialized treatment, it is necessary to expand public health insurance coverage of psychotherapy other than pharmacotherapy, such as cognitive-behavioral therapy, and increase reimbursement for such therapy.

The issue of consultation time has been prevalent in other countries as well, with Pollock et al. [28] noting in a study from the U.K. that “it is not simply a question of length of time, but the time psychiatrists spend in patient-centered listening is important in building a trusting relationship.” The similar demands of patients can be seen in the results of this study. It is required to provide medical care considering not only the length of time but also the quality of the treatment. In addition, decision support tools have been developed to facilitate communication between patients and psychiatrists in the absence of time and manpower, and include the use of letters, memos and the Internet [29–31]. As for the example in Japan, one of the authors participated in the creating the “question promotion pamphlet” [32] which can be used on digital as well as paper media. Ng et al. [33] stated that mobile mental health apps have potential benefits, but often do not use standardized assessment tools and in realty still have low utilization and sustained use. It is considered necessary to unify the use of validated evaluation tools in order to utilize them in clinical practice, and this seems to be an issue for the future.

The results of this survey suggest that it is necessary not to give up on the short consultation time as an unavoidable problem. In order to provide medical care that meets patient demands, it is necessary to make efforts to secure time in important situations, to listen to patients in a patient-centered manner, and to make use of the Internet and/or other tools for communication with patients. Psychiatrists struggle to secure consultation time; however, as their efforts are limited, reform of the medical treatment system itself is required.

### Individual responses

1. Criteria for selecting a psychiatrist

Other than statements regarding consultation time, “appropriate advice,” “listening,” “consideration for the patient and family’s position,” and “general explanation of medications” were cited, indicating the high level of patient interest in pharmacotherapy. Previous studies have reported that the length of psychiatric clinical experience of the treating psychiatrist and the duration of treatment by the same treating psychiatrist can be related to the degree of trust [34]. It has also been reported that the degree of patients’ trust in their psychiatrist and the relationship between the two may be related to their favorable attitude toward treatment, including pharmacotherapy, satisfaction with treatment, and the presence of consultation on medication [35, 36], which is similar to our findings.

Although it is difficult to address “proximity to home” only through efforts on the part of psychiatrist owing to relocation of psychiatrists, it is possible to incorporate “thinking about pharmacotherapy from the patient’s perspective” and “providing clear and detailed explanations rather than using medical jargon” into medical treatment.

2) Attitude of the psychiatrist in charge

Other than statements related to consultation time, “looking at the computer all the time,” “listening to me a little more,” and “making a medical report tailored to my physical condition at the time” were also mentioned. More severe responses than the in the survey employing Likert-style were observed, indicating that patients were dissatisfied.

The statement “looking at the computer all the time” indicates that patients are more concerned about whether or not their psychiatrists are seeing their faces. Keeping in mind that this point will lead to higher satisfaction, it is necessary to devise ways to improve patient satisfaction, such as placing the computer at an angle instead of in front of the psychiatrist, and attempting to look at the patient’s face whenever possible.

3) Communication skills of the psychiatrist

In addition to consultation time, other statements that were not included in the optional responses included “Does the doctor communicate with the patient to improve his or her psychological condition?,” “Does the doctor care about the parents?,” “Does the doctor have mood swings?,” and “Does the doctor try to build a trusting relationship?”.

In the open-ended question items, the respondents were asked “Please feel free to describe any other feelings you may have,” and were asked to write about topics other than the pre-listed options; therefore, it is assumed that descriptions about matters not included in the optional items, such as “psychological state of the patient,” “the mood of the psychiatrist in charge,” and “parents,” appeared. In particular, “the mood of the psychiatrist in charge” is something that patients are sensitive to, even if the psychiatrist himself does not pay attention to it. This is thought to include the patient’s anxiety about offending the psychiatrist in charge. In fact, it is necessary to keep in mind that the patients are highly sensitive to the psychological state of their psychiatrists during consultations.

Shiozawa et al. [37] stated that “attitudes and skills related to psychiatrist communication” and “consideration for patients to facilitate their own decisions” were associated with treatment satisfaction and medication adherence. Yamaguchi et al. [11] emphasized the importance of communication between patients and psychiatrists. They stated that good patient-psychiatrist relationship and communication may be associated with service satisfaction, attitude towards pharmacotherapy, patient truth-telling during consultations, and medication adherence [38, 39]. On the other hand, the level of evidence in the included studies is not high and rigorously designed studies need to be conducted.

Igarashi et al. [40] investigated the relationship between the frequency of consultations at psychiatric clinics, the duration of consultations, and patients’ satisfaction with their consultations. They found that “high-density visits” in which patients receive long counseling sessions in short intervals, are effective in busy Japanese psychiatric outpatient clinics.

An overview of these previous studies shows that many of the results are common to the factors “sufficient time,” “building trusting relationships,” and “explaining about medicines” that were derived from patients’ requests to open-ended items. Therefore, our interpretation may be taken as representing, to some extent, the thoughts of patients in Japan.

### Limitations of this study

Responses for this study were obtained from the “Questionnaire on Psychiatrists’ Examination, Attitude, and Communication Skills.”

The survey was conducted with the cooperation of the National Federation of Associations of Families with The Mental Illness in Japan and the Community Mental Health and Welfare Bonding; however, it is thought that the questionnaires were distributed through various other channels in addition to the secondary distribution from these organizations. The questionnaires were mainly distributed to family association members and their families (patients) in various regions; however, they were also distributed by them to patients and individuals other than family members. Therefore, we cannot identify all of the distribution sites, which is a limitation that prevents us from identifying the population of the survey.

Another possible limitation is that the survey was conducted by the first author, who is a psychiatrist, a patient, and a family member, from three different standpoints. Another limitation of the study is that it assumed that the survey results include not only the actual conditions of the respondents, but also their requests based on their actual conditions in their responses.

This study examined patients’ requests for psychiatrists’ consultation based on word–word associations of patients’ free responses with co-occurrence networks obtained using the KH Coder.

The interpretation of these co-occurrence relationships is based on the analyst’s perspective. However, one advantage of quantitative content analysis is that anyone can obtain the co-occurrence network shown in this study if they perform exactly the same operations using the KH Coder and the same data, making the results of this study highly reproducible.

As noted in the Background, the challenge is that studies using PROs are rarely large surveys and do not always use measures with a high level of validated evidence. The questionnaire of the survey we used in this study was created through discussions with patients and their families, and is not a validated scale. While there are advantages to an original questionnaire that emphasizes the patient or family perspective, the lack of comparability with other surveys should be considered.

## Conclusion

This study conducted quantitative content analysis on the reponses to open-ended question items in the questionnaire surevey of Japanese psychiatric patients. The results showed that patients concerned about whether psychiatrists spend enough time examining them and listen carefully to their indivisual demands.

Taking account of these consequences, it can be said that the open-ended question items analyzed in this study reflected the potentail patients’ demands toward psychiatrists. Also, both the usefulness of patients’open-ended responses to questionnaires and the effectiveness of their involvement (PPI) in the psychiatric research were suggested.

## Data Availability

The datasets used and/or analyzed in the current study are available from the corresponding author on reasonable request.

## Abbreviations

PPI: Patient and Public Involvement
PRO: Patient-reported outcomes

## Footnotes

1 These association and organization are the largest associations in Japan for psychiatric patients and their family.

2 The details of these closed-ended questions were shown in Natsukari et al. [18].

3 The phrases in parentheses and underlines written in the respondent’s citation added by the authors and same applies hereafter.
